# Cell cycle G2/M arrest through an S phase-dependent mechanism by HIV-1 viral protein R

**DOI:** 10.1186/1742-4690-7-59

**Published:** 2010-07-07

**Authors:** Ge Li, Hyeon U Park, Dong Liang, Richard Y Zhao

**Affiliations:** 1 Department of Pathology, Microbiology-Immunology, Institute of Human Virology, University of Maryland School of Medicine, Baltimore, MD, USA; 2 Lombardi Comprehensive Cancer Center, Georgetown University, Washington, DC, USA; 3 Department of Gene and Cell Medicine, The Black Family Stem Cell Institute, Mount Sinai School of Medicine, New York, NY, USA

## Abstract

**Background:**

Cell cycle G2 arrest induced by HIV-1 Vpr is thought to benefit viral proliferation by providing an optimized cellular environment for viral replication and by skipping host immune responses. Even though Vpr-induced G2 arrest has been studied extensively, how Vpr triggers G2 arrest remains elusive.

**Results:**

To examine this initiation event, we measured the Vpr effect over a single cell cycle. We found that even though Vpr stops the cell cycle at the G2/M phase, but the initiation event actually occurs in the S phase of the cell cycle. Specifically, Vpr triggers activation of Chk1 through Ser^345 ^phosphorylation in an S phase-dependent manner. The S phase-dependent requirement of Chk1-Ser^345 ^phosphorylation by Vpr was confirmed by siRNA gene silencing and site-directed mutagenesis. Moreover, downregulation of DNA replication licensing factors Cdt1 by siRNA significantly reduced Vpr-induced Chk1-Ser^345 ^phosphorylation and G2 arrest. Even though hydroxyurea (HU) and ultraviolet light (UV) also induce Chk1-Ser^345 ^phosphorylation in S phase under the same conditions, neither HU nor UV-treated cells were able to pass through S phase, whereas *vpr*-expressing cells completed S phase and stopped at the G2/M boundary. Furthermore, unlike HU/UV, Vpr promotes Chk1- and proteasome-mediated protein degradations of Cdc25B/C for G2 induction; in contrast, Vpr had little or no effect on Cdc25A protein degradation normally mediated by HU/UV.

**Conclusions:**

These data suggest that Vpr induces cell cycle G2 arrest through a unique molecular mechanism that regulates host cell cycle regulation in an S-phase dependent fashion.

## Background

Human immunodeficiency virus type 1 (HIV-1) viral protein R (Vpr) is a virion-associated accessory protein with an average length of 96 amino acids and a calculated molecular weight of 12.7 kDa [1]. Increasing evidence suggests that Vpr plays an important role in the viral pathogenesis of HIV-1. For example, infections with Vpr-defective viruses in rhesus monkeys, chimpanzees or human subjects seem to correlate with low viral load and slow disease progression [2-4], and some of the *vpr *point mutants could revert back to the wild type phenotype in the viral genome, which further supports the importance of Vpr in viral survival [5-7].

Vpr displays several distinct activities in host cells. These include cytoplasm-nuclear shuttling [4,8], induction of cell cycle G2 arrest [9], and cell killing [10]. The cell cycle G2 arrest induced by Vpr is thought to suppress human immune function by preventing T-cell clone expansion [11] and to provide an optimized cellular environment for maximal levels of viral replication [6]. Therefore, further understanding of Vpr-induced cell cycle G2 arrest could provide additional insights into the molecular actions of Vpr in augmenting viral replication and modulation of host immune response.

Progression of cell cycle from G2 phase to mitosis requires activation of the cyclin-dependent kinase 1 (Cdk1), which determines onset of mitosis in all eukaryotes [12-14]. Cdk1 is typically phosphorylated on Tyr15 by Wee1 kinase in late G2 [13,15], and it is rapidly dephosphorylated at the same amino acid residue by the Cdc25 tyrosine phosphatases to trigger entry into mitosis [16]. Thus it is the balance between the Wee1 kinase and Cdc25 phosphatases activities that determines cellular entry of mitosis. In human cells, there are three Cdc25 homologues, Cdc25A, Cdc25B and Cdc25C [17]. Cdc25A plays general roles in regulating cell-cycle transition, especially in G1/S transition and the exit of mitosis [18]. The activity of Cdc25A is tightly regulated at the protein level, being periodically synthesized and degraded *via *ubiquitin-mediated proteolysis [19]. Cdc25A is rapidly degraded in response to DNA damage or stalled replication and is known to be a crucial substrate in the mitotic DNA checkpoint response [20,21]. Ultraviolet light (UV) or hydroxyurea (HU) treatments are known to rapidly activate the ATR-Chk1 pathway, leading to phosphorylation of Cdc25A and triggering the signal for its degradation by proteasome leading to S-phase arrest [20]. On the other hand, Cdc25B and Cdc25C have a more restricted role in promoting progression from G2 phase to mitosis [18]. Despite the seemingly similarity in functions, however, Cdc25B and Cdc25C have distinct roles temporally in cell proliferation with Cdc25B activity peaking before Cdc25C [22,23]. Cdc25B may acts as a 'starter phosphatase', promoting the initial activation Cdk1-cyclinB, which in turn initiates mitosis through the up-regulation of Cdc25C [24]. Deletion of all Cdc25 genes is lethal. Depletion of any one of these two phosphatases will result in significant delay of mitotic entry; however, this will not lead to cell cycle G2 arrest due to the functional redundancy of the Cdc25 phosphatases [25,26]. In response to DNA damage such as double strand DNA breaks (DSBs), Cdc25C is phosphorylated on Ser216 *via *a Chk1/Chk2-mediated pathway then is bound to 14-3-3, leading to the translocation of Cdc25C from the nucleus to the cytoplasm for final proteasome-mediated protein degradation, leading to cell cycle G2/M arrest [27,28]. Previous studies demonstrated that Vpr induces cell cycle G2 arrest through the promotion of hyper-phosphorylation of Cdk1 [9,29,30], which is achieved through inhibition of the Cdc25 phosphatase [31-34] and the activation of the Wee1 kinase [32,33].

Eukaryotic cells have an elaborate network of checkpoints to monitor the successful completion of every cell cycle step and to respond to cellular abnormalities such as DNA damage and replication inhibition as they arise during cell proliferation. Among many of the checkpoint control regulations, ATR or ATM and Chk1 or Chk2 are essential kinases of cell cycle checkpoint controls [35,36]. For example, treatment of cells with UV or HU causes single strand break (SSB) or disruption of DNA replication respectively, which triggers DNA replication checkpoint through activation of the sensor kinase ATR. Activated ATR in turn results in the specific phosphorylation and activation of the effector kinase Chk1 at the Ser^345 ^residue leading to the S-phase arrest. Similarly, when severe DNA damage such as DSBs is induced by ionizing radiation (IR), DSB signals mostly activate the sensor kinase ATM, which in turn activates the effector Chk2 kinase leading to cell cycle G2 arrest [31,37-39]. However, both Chk1 and Chk2 can phosphorylate three Cdc25 homologues to induce cell cycle S or G2 arrest under different circumstances [40,41].

Given that the DNA damage checkpoint and Vpr both induce G2 arrest through inhibitory phosphorylation of Cdk1, Vpr might induce G2 arrest through the DNA damage checkpoint pathway. Indeed, Tachiwana *et al. *showed Vpr induces DNA DSBs, which supports the idea that Vpr induces G2 arrest through DNA damage checkpoint [42]. However, a different report showed that Vpr does not induce DNA DSBs [43]. Moreover, the ATR kinase instead of the ATM kinase was found to play a major role in Vpr-induced G2 arrest through the phosphorylation and activation of Chk1 [44,45]. These studies suggested that Vpr-induced G2 arrest may in fact resemble more the activation of DNA replication checkpoint than the DNA damage checkpoint control. Further studies have shown numerous similarities between the ATR pathway activated by Vpr and that by HU/UV. These similarities include the requirement for Rad17 and Hus1, the induction of phosphorylation on Chk1 and the formation of nuclear foci by RPA, 53BP1, BRCA1 and γH2AX [43-45]. However, these conclusions remain controversial based on the fact that expression of *vpr *does not change the radiosensitivity of the checkpoint defective mutants [46] and/or increase gene mutation frequency [47], which argues against the possibility that Vpr actually causes DNA damage for G2 induction. Furthermore, activation of DNA replication checkpoint generally leads to S phase arrest, but not G2 arrest. In another study, by using siRNA, a special isoform of PP2A was shown to play an essential role in the G2 arrest induced by Vpr in human cells. Unlike UV/HU-induced Chk1-Ser^345 ^phosphorylation, the phosphorylation of Chk1-Ser^345 ^induced by Vpr required the existence of this PP2A, but was independent of γH2AX activation [48]. This finding suggests that Vpr-induced G2 arrest may be different to a certain extent from the DNA damage and replication checkpoint controls.

Even though Vpr-induced cell cycle G2 arrest has been extensively studied, what triggers the cell cycle G2 arrest by Vpr is at present unknown. One of the technical difficulties to examine this molecular event is that most of the early studies on Vpr-induced G2 arrest measured the Vpr effect 48-72 hours after the introduction of Vpr into asynchronized cell populations. To facilitate this study, measurement of the initiating event(s) for Vpr-induced G2 arrest would benefit from a system that uses synchronized cells and minimizes the time between initiation of Vpr expression and the measurement of the G2 arrest. For this reason, we have adapted an approach that allows us to monitor the cellular signaling for Vpr-induced G2 arrest within a single cell cycle. By using this single cell cycle assay, we have now uncovered that the G2-initiating signal(s) induced by Vpr is actually generated in the S phase of the cell cycle through induction of Chk1-Ser^345 ^phosphorylation. To the best of our knowledge, the Vpr effect described here is unique and may represent a novel viral action for modulating host cell cycle regulation.

## Results

### Vpr-induced Chk1 Activation Occurs in the S Phase of the Cell Cycle

**Figure 1:**
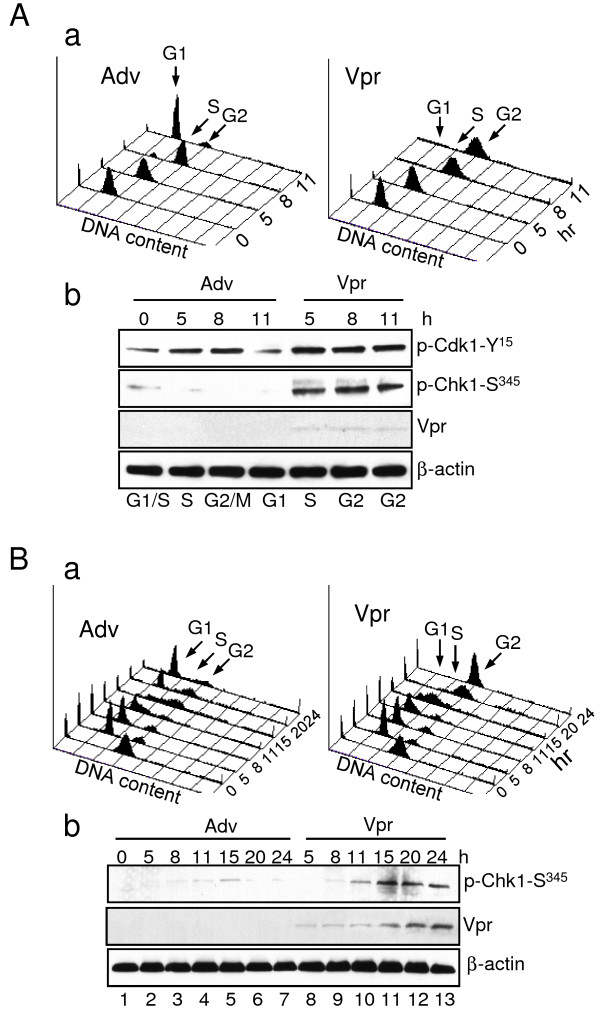
**Vpr induces cell cycle G2/M arrest through activation of Chk1 *via *Ser**^**345 **^**phosphorylation in S phase of the cell cycle**. **A**. HeLa cells synchronized at the G1/S boundary by double thymine (DT) block were transduced with Adv control or Adv-Vpr (MOI 1.0) and released from the block at time 0. The cell cycle profiles measured by DNA content (**a**) were detected from time 0 to 11 hours after the DT release. The Cdk1-Tyr^345 ^or Chk1-Ser^345 ^phosphorylation status (**b**) were detected in the Vpr-positive or Vpr-negative cells collected at indicated time. **B**. HeLa cells, which were first synchronized in M phase by Nocodazole (100 ng/ml), were transduced with Adv or Adv-Vpr and detected the same way as shown in (**A**). Note that very weak Vpr was detected in (**A-b**) because Ad-Vpr was only transduced within 5 to 11 hours. The Vpr protein was clearly detected subsequently at about 15 hours after viral transduction (**B-b**).

To monitor the initiating event of cellular signaling for Vpr-induced G2 arrest, we adopted a single cell cycle assay to measure this event in a synchronized cell population. Specifically, HeLa cells were firstly synchronized at the G1/S boundary of the cell cycle by the double thymidine (DT) block as described previously [49]. Synchronized HeLa cells were then transduced immediately after released from the DT block with an adenoviral vector control (Adv) or a *vpr*-carrying adenoviral vector (Adv-Vpr) at a multiplicity of infection (MOI) of 1.0. Cells were collected at the indicated time after transduction, and cell cycle profiles were monitored by flow cytometric analysis. As shown in Figure 1A-a, >90% of cells were observed in G1 when the synchronized cells were released from the DT treatment (0 hr). Without Vpr, cells progressed to S phase by 5 hours, G2/M by 8 hours and returned back to the G1 phase by 11 hours (Figure 1A-a, left). Similar cell cycle progression was also observed in cells expressing *vpr *in the first 8 hours. However, cell cycling stopped at the G2/M phase by 11 hours (Figure 1A-a, right). Vpr-induced G2 arrest was further confirmed by the elevated phosphorylation of Cdk1-Tyr^15 ^as measured by Western blot analysis (Figure 1A-b, top row). Please note that the entire cell cycle takes longer than 11 hours to complete typically around 22-24 hours. The 11 hours after release of the DT block is the shortest time within a single cell cycle that we could measure Vpr-induced G2 arrest.

Since previous studies showed that the Chk1-S^345 ^phosphorylation is required for Vpr-induced G2 arrest [44,48], potential Chk1-S^345 ^phosphorylation was measured as a marker for Vpr-induced G2 arrest by Western blot analysis. Consistent with the idea that Chk1 activation, as indicated by Chk1-Ser^345 ^phosphorylation, triggers G2 induction [44,48], the Chk1-Ser^345 ^phosphorylation appeared as early as 5 hours (in S-phase) after Adv-Vpr transduction (Figure 1A-b, second row). In contrast, no Chk1-Ser^345 ^phosphorylation was observed in the Adv transduction control. To further verify this finding and test whether the activation of Chk1 induced by Vpr indeed starts in S phase, HeLa cells were synchronized in the M phase (Figure 1B) by treatment with 100 ng/mL of Nocodazole [50]. Cell cycle profiles and Chk1-Ser^345 ^phosphorylation were then detected. If Vpr-induced Chk1 activation is S-phase independent, Chk1-Ser^345 ^phosphorylation would be observed within 5 hours after viral transduction regardless of the cell cycle stages. In contrast, if Vpr-induced Chk1 activation is S-phase dependent, Chk1-Ser^345 ^phosphorylation would not be observed until the transduced cells have entered the next S phase. As shown in Figure 1B-b, first row, no Chk1-Ser^345 ^phosphorylation was detected until 11 hours after Adv-Vpr viral transduction when cells entered the S phase, which precedes the G2 arrest. Consistently, no G2 cell accumulation was observed prior to Chk1-Ser^345 ^phosphorylation. However, after the cells passed through the S phase at 15 hours, the cells stopped at the next G2 phase at 20 hours, whereas the Adv-transduced control cells continued into the G1 phase (Figure 1B-a). Together, these data suggest that Vpr triggers the activation of Chk1, as shown by Chk1-Ser^345 ^phosphorylation, in the S-phase of the cell cycle.

### Chk1-Ser^345 ^Phosphorylation Is Exclusively Required for Vpr-induced G2 Arrest

**Figure 2:**
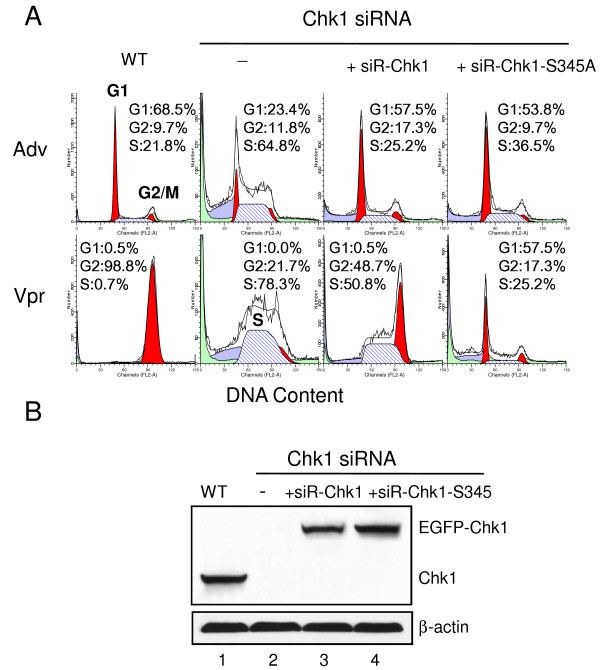
**Chk1-Ser**^**345 **^**is exclusively required for Vpr-induced G2 arrest**. HeLa cells were first transfected with wild type (WT) siRNA-resistant pEGFP-Chk1 (siR-Chk1) or pEGFP-Chk1 Ser345A mutant (siR-Chk1-S345A) plasmids. The endogenous Chk1 mRNA was then depleted by a specific Chk1 siRNA for 24 hrs followed by Adv or Adv-Vpr transduction. The symbol "+" indicates presence of the siR-Chk1 or siR-Chk1-S345A plasmids. The dash sign "-"means no plasmid was introduced in wild-type Chk1, depleted by siRNA. The cell cycle profiles of the indicated cell lines were measured 48 hours after the adenoviral transduction by flow cytometric analysis (**A**). Expression of endogenous or siRNA-resistant Chk1 constructs from indicated cell lines was confirmed by Western blot analysis using anti-Chk1 antibody at the same time of flow cytometric analysis (**B**). Note that the siR-Chk1 or siR-Chk1-Ser345A gene products cannot be depleted by the normal "Chk1 siRNA" used here because silent mutations were incorporated into the *Chk1 *genes during site-directed mutagenesis. These silent mutations will not alter the intended protein sequences, i.e., wild type Chk1 or Chk1-Ser345A.

Our data and other early reports have demonstrated that the activation of Chk1 is required for Vpr-induced G2 arrest, and Chk1 was shown to be hyper-phosphorylated at the Ser^345 ^residue with *vpr *gene expression [44,48]. However, there was no direct evidence that Ser^345 ^phosphorylation of Chk1 is exclusively required for Vpr-induced G2 arrest. To test whether Chk1-Ser^345 ^phosphorylation is indeed required for Vpr-induced G2 arrest, the Ser residue of Chk1 at 345 was converted to Ala on the pEGFP-Chk1 plasmid by use of site-directed mutagenesis. To allow specific depletion of the endogenous *chk1 *gene without interfering with the plasmid *chk1 *gene expression, siRNA-resistant wild type Chk1 (siR-Chk1) or Ser345A (siR-Chk1-S345A) mutant Chk1 genes were constructed. These were achieved by introducing synonymous nucleotide mutations at the third codons of the siRNA-targeting site, which result in silent mutations that will not affect the normal protein sequences, but they cannot recognized by the normal siRNA we used to deplete endogenous Chk1. In this configuration, possible requirement of Chk1-Ser^345 ^phosphorylation for Vpr-induced G2 arrest could be demonstrated specifically either in the Chk1-depleted cells or with expression of a *chk1*-S345A mutant plasmid; whereas re-introduction of the siRNA-resistant wild type of *chk1 *plasmid into the Chk1-depleted cells should restore Vpr-induced G2 arrest. As shown in Figure 2A, depletion of *chk1 *resulted in S phase accumulation as reported previously [51,52]. Re-introduction of the siRNA-resistant wild type of *chk1 *plasmid into the Chk1-depleted cells indeed restored Vpr-induced G2 arrest. Moreover, expression of the mutant *chk1*-S345A plasmid in the Chk1-depleted cells failed to restore G2 arrest. Successful deletion of endogenous Chk1 and expression of EGFP-Chk1 fusion proteins were further confirmed by Western blot analysis (Figure 2B). Thus these data support the idea that Chk-Ser^345 ^phosphorylation is specifically required for Vpr-induced G2 arrest.

### Chk1-Ser^345 ^Is Activated by HU, UV and Vpr with Different Cell Cycle Outcomes

**Figure 3:**
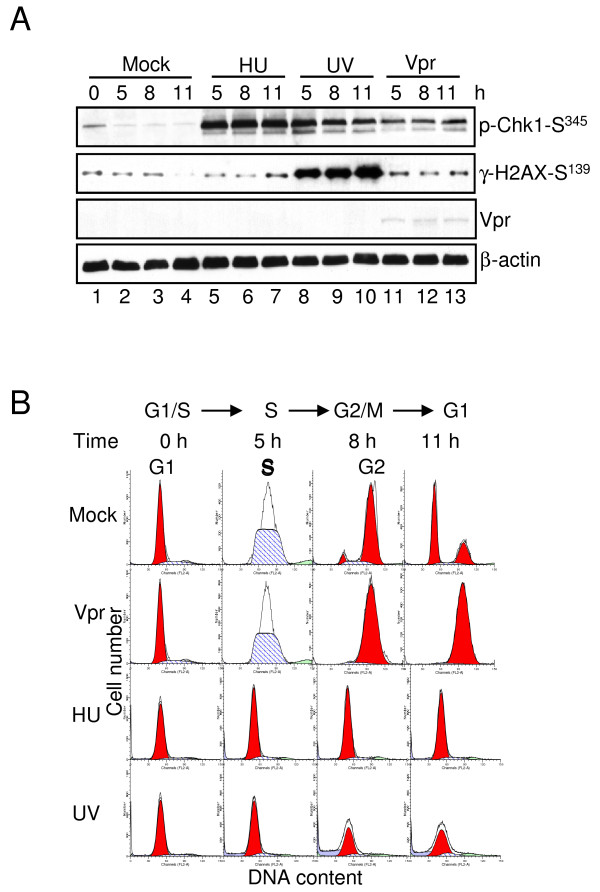
**Chk1-Ser**^**345 **^**is activated by Vpr and HU/UV with different cell cycle outcomes**. Synchronized G1/S HeLa cells by DT were treated with HU, UV or transduced with Adv-Vpr at time 0, collected at the indicated time, and then subjected to Western blot analysis (**A**) using anti-Chk1-Ser^345 ^and anti-γH2AX-Ser^139 ^antibodies. The cell cycle profiles of differently treated cells were analyzed at the indicated time after the DT release by flow cytometric analysis (**B**).

Chk1 is activated by Vpr in S phase 5 hours after the DT release, suggesting Chk1 might be a key regulatory factor to trigger Vpr-induced G2 arrest in S phase. However, other genotoxic agents such as UV and HU have also been shown to activate Chk1-Ser^345 ^phosphorylation and to trigger DNA replication checkpoint controls [53,54]. To compare the potential difference in cell cycle outcome when Vpr or HU/UV induces Chk1-Ser^345 ^phosphorylation, synchronized G1/S HeLa cells were first treated with HU, UV or Adv-Vpr transduction and then collected at 5, 8, and 11 hours after treatment, respectively. The harvested cells were then subjected to cell cycle analysis and Western blot analyses using anti-Chk1-Ser^345 ^and anti-γH2AX-Ser^139 ^antibodies. As shown in Figure 3A (first row), Chk1-Ser^345 ^phosphorylation was observed as early as 5 hours after the DT release in all three treatments. Strong γH2AX-Ser^139 ^phosphorylation was also observed in the UV-treated cells (Figure 3A, second row, lanes 8-10) as early as 5 hours after treatment as previously described [55]. In contrast, only background level of γH2AX-Ser^139 ^phosphorylation was found in *vpr*-expressing and HU-treated cells (Figure 3A, second row, lanes 5-7 and lanes 11-13). Cell cycle profiles were monitored by flow cytometric analysis. While untreated cells had normal cell cycle progression, *vpr*-expressing cells stopped at the G2 phase of the cell cycle; however, neither HU nor UV-treated cells were able to pass through S phase. They both arrested at the G1/S boundary of the cell cycle during the entire experimental period. Therefore, even though HU, UV and Vpr all induce Chk1-Ser^345 ^phosphorylation, the outcomes are quite different, implicating that the activated Chk1 may trigger different downstream events leading to G1/S or G2 arrests, respectively.

### HU/UV Promotes Proteasome-mediated Protein Degradation of Cdc25A

**Figure 4:**
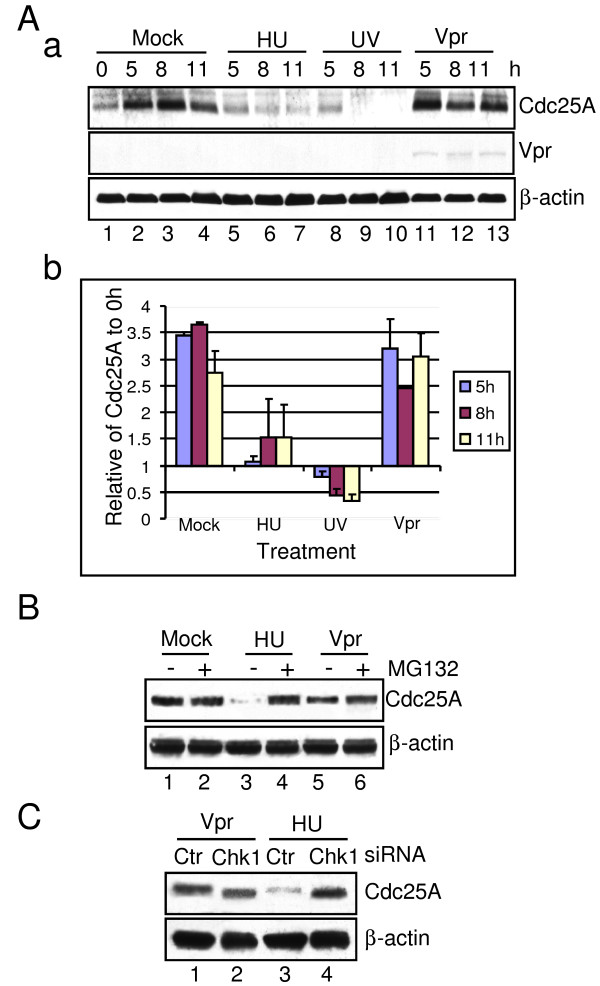
**Vpr has little or no effect on proteasome-mediated protein degradation of Cdc25A in contrast to HU/UV**. **(A) **Synchronized G1/S HeLa cells treated with HU, UV or transduced with Adv-Vpr were collected at the indicated time, and then subjected to Western blot analysis using anti-Cdc25A and anti-Vpr antibodies (**a**). β-actin was used as a loading control. The relative intensity of the Cdc25A protein levels to β-actin was determined by densitometry and the Cdc25A protein level at 0 hour was set as 1.0. (**b)**. The results presented are the average of three independent experiments. (**B) **Synchronized HeLa cells were treated with 50 μm MG132 at 0 hour and collected 5 hours after treatment. The protein level of Cdc25A was detected by Western blot analysis. (**C) **HeLa cells were pre-treated with specific siRNA against Chk1, which were then synchronized at G1/S boundary by the DT blocks. HU- or Vpr-treated cells were collected 5 hours after the DT release. The protein level of Cdc25A was detected by Western blot analysis using the indicated antibodies.

One of the downstream events driven by activated Chk1 is the inhibitory phosphorylation of Cdc25 phosphatases. Since all three Cdc25 homologues are the essential substrates of Chk1 during DNA damage/replication checkpoints, which one of the three Cdc25s is being inactivated by Chk1 could define the cell cycle outcome [17,20,21]. Previous studies suggested that Cdc25A is one of direct targets of activated Chk1, which results in the S phase arrest when cells are challenged by HU or UV [20,21]. To determine whether Cdc25A is affected by Vpr or whether it contributes to the observed differences of cell cycle profiles in cells treated with HU/UV or Vpr (Figure 3B), synchronized G1/S HeLa cells were prepared by DT block and treated with HU, UV or Adv-Vpr transduction as described above. The Cdc25A protein levels collected over time were then detected by Western blot analyses using an anti-Cdc25A antibody. As shown in Figure 4A-a, first row, and Figure 4A-b, Cdc25A protein level in a normal cell cycle rose significantly from G1/S (0 hr) to S (5 hours) and reached maximum in the G2 phase (8 hours) followed by a small decrease in G1 phase (Figure 4A-a, first row, lanes 1-4). Similar to normal cells, relatively high levels of Cdc25A, with a small dip in the G2 phase, were seen from S phase to G1 phase in the *vpr*-expressing cells (Figure 4A-a, first row, lanes 11-13). Since the Cdc25A protein profile in *vpr*-expressing cells showed similar pattern to normal cells, it suggests that Vpr has little or no effect on Chk1-mediated Cdc25A protein production or degradation. In contrast to this pattern observed in normal and *vpr*-expressing cells, much reduced Cdc25A proteins were observed in cells treated with HU or UV throughout the cell cycle (Figure 4A-a, first row, lanes 5-10). To test whether the low Cdc25A protein levels observed in the HU/UV-treated cells are due to prevention of protein production or promotion of proteasome-mediated protein degradation, the protein levels of Cdc25A were further compared between cells treated with the proteasome inhibitor MG132 (50 μM) and untreated control cells (Figure 4B; only cells treated with HU and collected 5 hours after the DT release are shown here as control). While the normal Cdc25A protein level was completely restored in HU-treated cells treated with MG132, only a small and non-appreciable increase of Cdc25A was noted in the *vpr*-expressing cells treated with MG132. These data suggest that HU/UV promotes protein degradation of Cdc25A through a proteasome-mediated mechanism. Similarly, these data further confirmed that, unlike UV or HU, Vpr has little, if any, impact on the Cdc25A protein level in these cells.

To ascertain the observed differences in the Cdc25A protein levels are indeed due to Chk1 activation, Chk1 was depleted by specific siRNA against Chk1, or by control siRNA. As shown in Figure 4C, depletion of Chk1 completely abolished HU-mediated Cdc25A degradation (Figure 4C, lanes 4 *vs*.3). In contrast, depletion of Chk1 had no obvious effect on Cdc25A protein level in *vpr*-expressing cells (Figure 4C, lanes 2 *vs*. 1). Note that the Cdc25A protein bands migrated a little faster in Chk1-depleted cells than that in control cells (Figure 4C, lanes 2 *vs*. 1; lanes 4 *vs*. 3). This difference in protein size could potentially be due to the lack of Cdc25A phosphorylation by Chk1 as previously described [20,21]. Together, these data suggest that, in contrast to Vpr, HU/UV promotes protein degradation of Cdc25A through Chk1.

### Vpr Promotes Proteasome-mediated Protein Degradations of Cdc25C and Cdc25B

**Figure 5:**
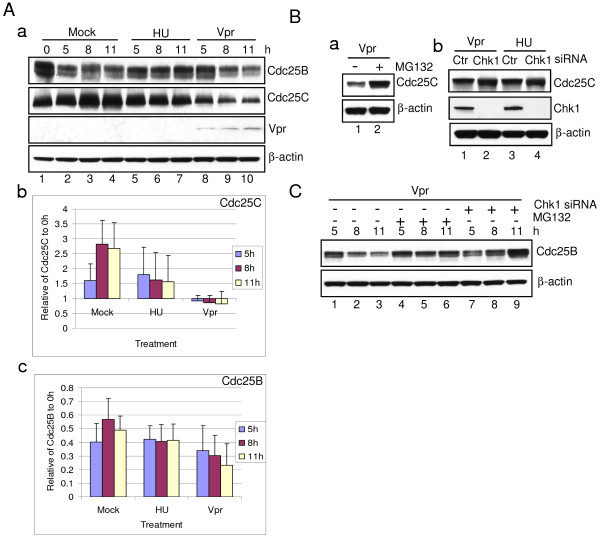
**Vpr promotes proteasome-mediated protein degradation of Cdc25B and Cdc25C**. (**A) **Synchronized G1/S HeLa cells treated with HU or transduced with Adv-Vpr were collected at indicated time, and then subjected to Western blot analysis using anti-Cdc25B or anti-Cdc25C antibody, respectively (**a)**. β-actin was used as a loading control. The relative intensity of the Cdc25B (**b**) or Cdc25C (**c**) protein levels to β-actin were determined by densitometry. The results presented are the average of three independent experiments. (**B) **Synchronized HeLa cells were pre-treated with specific siRNA against Chk1 or treated with 50 μm MG132 at 0 hour and collected at the indicated time. The protein level of Cdc25B was detected by Western blot analysis. (**C) **Synchronized HeLa cells were treated with 50 μm MG132 at 0 hour and collected 11 hours after treatment. The protein level of Cdc25C was detected by Western blot analysis (**a)**. HeLa cells were pre-treated with specific siRNA against Chk1, which were then synchronized at G1/S boundary by DT treatment. HU or Vpr treated cells were collected 11 hours after DT release. The protein level of Cdc25C was detected by Western blot analysis using indicated antibodies (**b**).

Since Vpr had little or no effect on Chk1-mediated Cdc25A protein degradation, we next examined whether Cdc25B/C are the substrates of Chk1 for Vpr to induce G2 arrest. Like that described above, the synchronized G1/S HeLa cells were treated with HU/UV or Adv-Vpr transduction, and the Cdc25B/C protein levels were compared by Western blot analyses using anti-Cdc25B or anti-Cdc25C antibodies. As shown in Figure 5A-a, second row, and Figure 5A-b, significant and gradual increases of Cdc25C were observed in the normal cells from 0 to 8 hours (Figure 5A-a, second row, and Figure 5A-b). HU-treatment resulted in small but perhaps insignificant decrease of Cdc25C over time; in contrast, Vpr induced a rather strong reduction of Cdc25C over time (Figure 5A-a, second row, lanes 8-10). To ascertain Vpr-mediated reduction of Cdc25C protein levels is also through proteasome-mediated proteolysis, cells were treated with the proteasome inhibitor MG132, the Chk1-specific siRNA or were untreated. Depletion of Chk1 restored the protein level of Cdc25C (Figure 5B-b, lanes 2 *vs*. 1). Normal protein level of Cdc25C was also seen when the same cells were treated with MG132 (Figure 5B-a). The depletion of Chk1 by siRNA was confirmed by Western blot analysis (Figure 5B-b, row 2).

There were also overall small but appreciable decreases of Cdc25B in *vpr*-expressing cells at all three time points as soon as cells were released from the DT block (Figure 5A-a, first row, lanes 8-10 *vs*. lane 1). This was in contrast to the normal and HU-treated cellular profile of Cdc25B, where a small increase of Cdc25B was seen instead (Figure 5A-a, first row, lanes 8-10 *vs*. lanes 2-4); while HU-treated cells remained constant (lanes 5-7). Like Cdc25C, the treatment of *vpr*-expressing cells with MG132 restored normal level of Cdc25B, suggesting that the observed reduction of Cdc25B was indeed due to degradation by the proteasome (Figure 5C, lanes 4-6). Moreover, Vpr-induced reduction of Cdc25B is likely mediated through Chk1 since the depletion of Chk1 by siRNA also restored the protein level back to the normal level (Figure 5C, lanes 7-9). Altogether, these results support the idea that Vpr promotes proteasome-mediated protein degradation of Cdc25C and possibly Cdc25B through Chk1.

### Vpr Promotes Chk1-Ser^345 ^Phosphorylation and G2 Arrest Possibly through Signaling of DNA Re-replication via Cdt1

**Figure 6:**
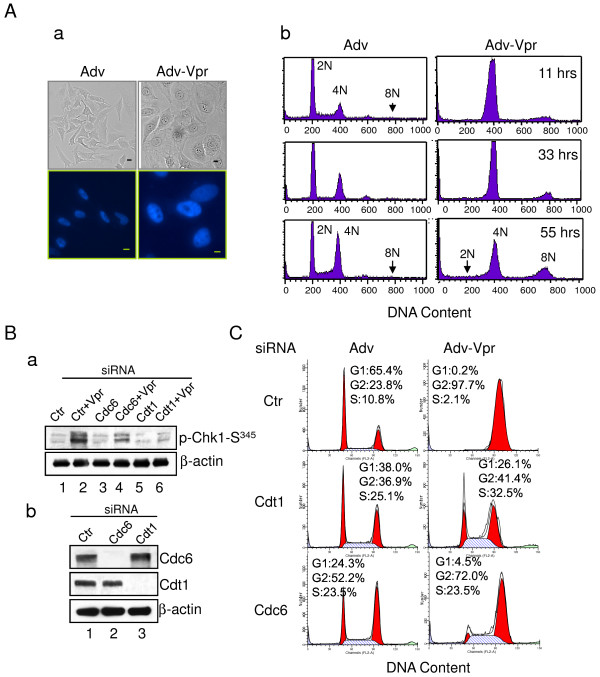
**Possible roles of Cdt1 and Cdc6 in Vpr-induced Chk1-Ser**^**345 **^**phosphorylation and G2 arrest in HeLa cells**. **(A) a**. Vpr induces cellular gross enlargement (top) with single enlarged nuclei (bottom). HeLa cells were synchronized in G1/S as described. Cells were then stained with DAPI. Images were captured 11 hours after Vpr transduction using a Leica DMR fluorescence microscope (DM4500B; Leica Microsystems) equipped with a high-performance camera (Hamamatsu) under visual light (top) and fluorescence (bottom). Scale bar: 10 μm. **b**. Vpr promotes the accumulation of DNA polyploidy as indicated by presence of 8N DNA. HeLa cells were synchronized in G1/S as described. DNA ploidy was measured by propidium iodide staining using flow cytometric analysis over time. (**B**) Synchronized G1/S HeLa cells, treated with Cdc6, Cdt1 or control siRNA, were transduced with Adv-Vpr at time 0 and then collected at 5 hours after viral transduction. The cell lysates were subjected to Western blot using anti-Chk1-Ser^345 ^antibody (**a**). The knockdown efficiency of Cdc6 or Cdt1 siRNA was verified by using anti-Cdc6 or anti-Cdt1 antibody with β-actin as controls (**b**). (**C**). Synchronized G1/S HeLa cells, treated with Cdc6, Cdt1 or control siRNA, were transduced with Adv or Adv-Vpr at time 0 and then collected at 11 hours after viral transduction for flow cytometric analysis. Ctr, control.

Since the G2-inducing signal appears to be generated in S phase of the cell cycle, one possibility is that Vpr could potentially interfere with DNA synthesis either by blocking DNA replication or by interfering with DNA replication. In eukaryotes, DNA synthesis is strictly regulated by DNA replication licensing factors Cdt1 and Cdc6 which serve to ensure that DNA replicates only once per cell cycle [56,57]. Typically, in late G1 phase, Cdt1 is activated by binding of Cdc6 to promote formation of pre-replication complexes [58]. Upon the start of DNA replication, Cdt1 is rapidly inhibited or degraded by various mechanisms to prevent re-replication (for a recent review, see [59]). However, when Cdt1 and Cdc6 are improperly elevated, DNA re-replication occurs, which causes Chk1-Ser^345 ^phosphorylation [60]. Previous studies suggested that some HIV-infected cells increase cellular DNA ploidy [61], and Vpr promotes aneuploidy [62]. As *vpr*-expressing cells are obviously capable of passing through the S phase, Vpr-induced aneuploidy suggests that Vpr could either cause DNA-replication [57,63], which should occur within a single nucleus of a cell, or failed cytokinesis for which multiple nuclei should be seen in a single cell. To test these possibilities, we first compared the cellular and nuclear morphologies of HeLa cells between Adv-control and Adv-Vpr expressing cells 11 hours after adenoviral transduction. As shown in Figure 6A-a (top), the Vpr-producing cells were grossly enlarged in comparison with the Adv-control cells as described previously [64]. Nuclear staining with propidium iodide (PI) showed much larger cells with a single nucleus in each of the Vpr-producing HeLa cells when compared to control cells (Figure 6A-a, bottom). These observations suggest that Vpr may induce DNA re-replication within a single nucleus of an individual cell instead of inducing failed cytokinesis.

To further examine whether we could actually observe the accumulation of DNA polyploidy over time, DNA content in the Adv-control and Adv-Vpr transduced cells was measured by flow cytometric analysis over a period of 55 hours. As we have shown in Figure 3B, most of the synchronized HeLa cells returned back to G1 stage (2N) by 11 hours after the DT release, with a minor amount of cells being in G2/M (4N) (Figure 6A-b). The % of G2 cells gradually increased over time from 33 to 55 hours. In contrast, nearly 100% of the *vpr*-expressing cells were arrested in the G2 phase by 11 hours after the DT release with no visible G1 cells (Figure 6A-b). A small hump of 8N cells was seen at 11 hours. This 8N population appeared to increase over time as the 4N cell population decreased (Figure 6A-b). All together, these findings indicate that Vpr promotes DNA re-replication, but at a relatively low level.

To further test whether Vpr could potentially affect the Cdt1 or Cdc6 activity leading to Chk1-Ser^345 ^phosphorylation, either one or both proteins were depleted using specific siRNA against Cdt1 and/or Cdc6. As shown in Figure 6B-a, untreated HeLa cells showed basal level phosphorylation of Chk1-Ser^345^. Consistently, cells transduced with Adv-Vpr showed strong phosphorylation of Chk1-Ser^345 ^even with pretreatment of control siRNA (Figure 6B-a, lane 2). While depletion of Cdt1 or Cdc6 had no obvious effect on Chk1-Ser^345 ^phosphorylation (Figure 6B-a, lanes 3 and 5), interestingly, the depletion of Cdt1 significantly reduced Chk1-Ser^345 ^phosphorylation induced by Vpr (Figure 6B-a, lane 6). Reduced Chk1-Ser^345 ^phosphorylation was also seen in the *vpr*-expressing and Cdc6-depleted cells, but the latter showed less reduction than that from Cdt1 depletion (Figure 6B-a, lane 4 *vs*. 6). Double depletion of Cdt1 and Cdc6 showed no additional reduction on the Vpr effect (data not shown). The successful depletion of Cdt1 or Cdc6 protein by siRNAs was confirmed by Western blotting with antibody against Cdt1 or Cdc6 (Figure 6B-b).

To evaluate whether the effect of Vpr on Cdt1 or Cdc6 contributes to Vpr-induced G2 arrest, we tested the same Cdt1 or Cdc6 depletion effect on Vpr-induced G2 arrest using the single cell cycle assay 11 hours after the DT release and Adv-Vpr transduction. Consistent with cell cycle profiles shown in Figure 1A, about 65% of the synchronized cells returned back to the G1 phase by 11 hours; in contrast, about 98% of Adv-Vpr transduced cells arrested at the G2/M phase (Figure 6C, top panel). Significantly, a strong reduction of G2 cells (from 98% to 41%) was observed in the Cdt1-depleted cells; a relative small reduction (from 98% to 72%) was also observed in the Cdc6-depleted cells. Noticeably, depletion of either Cdc6 or Cdt1 alone slightly increased the G2 cell populations. However, such a G2 increase would only underestimate the reduction of Vpr-induced G2 arrest in the Cdt1- or Cdc6-depleted cells. Together, these data suggest that Vpr promotes low level of DNA re-replication through Cdt1 and with a lesser extent through Cdc6, which triggers Chk1-Ser^345 ^phosphorylation possibly leading to cell cycle G2 arrest.

**Figure 7:**
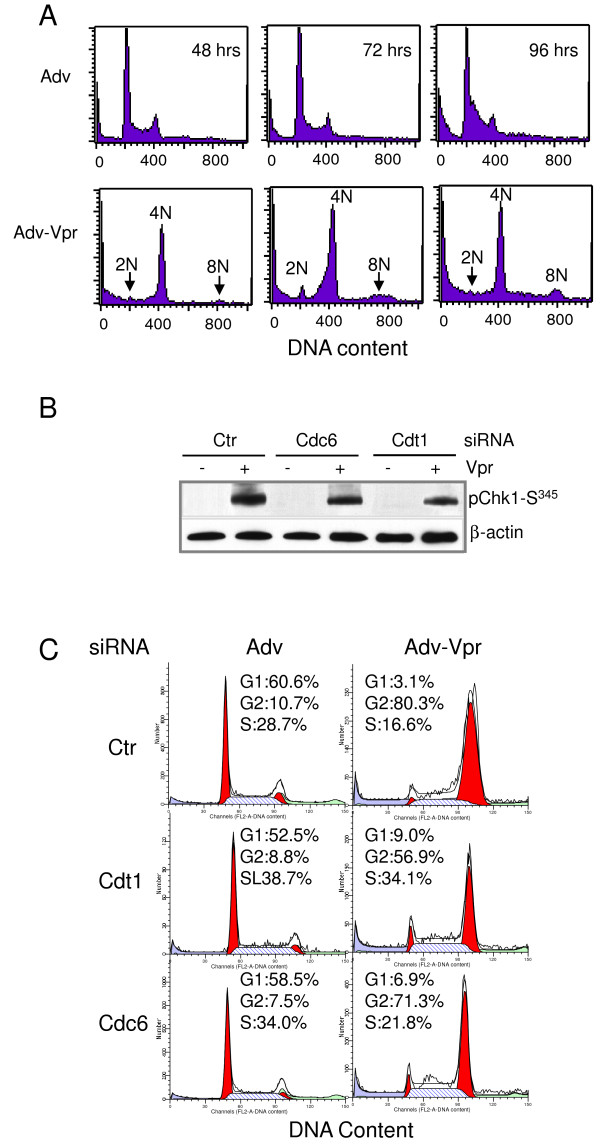
**Possible roles of Cdt1 and Cdc6 in Vpr-induced Chk1-Ser**^**345 **^**phosphorylation and G2 arrest in CEM-SS cells**. **(A) **Vpr promotes accumulation of DNA polyploidy as indicated by the presence of 8N DNA. Asynchronized CEM-SS cells were grown under the normal cell culture condition, and transduced with Adv viral control or Adv-Vpr. Cells were collected at indicated time point and DNA ploidy was measured by PI staining using flow cytometric analysis. (**B**) Asynchronized CEM-SS cells were pretreated with Cdt1, Cdc6 or control (Ctr) siRNA, and then transduced with Adv or Adv-Vpr 24 hours after addition of siRNAs. Cells were then harvested 48 hours post-transduction. The cell lysates were subjected to Western blot using anti-Chk1-Ser^345 ^antibody. The knockdown efficiency of Cdc6 or Cdt1 siRNA was verified by using anti-Cdc6 or anti-Cdt1 antibody with β-actin as protein loading controls. (**C**). CEM-SS were treated the same way as described in (**B**). The cells were harvested 48 hours post-transduction and the cell lysates were then subjected to flow cytometric analysis.

HeLa cells are not the natural target cells of HIV-1. In addition, HeLa cells are immortalized with human papillomavirus virus 18 which encodes viral proteins that may interfere with cell cycle regulation. To see whether the same effects of Vpr on the DNA re-replication, Chk1-Ser^345 ^phosphorylation and Cdt1 as well as Cdc6 could be observed in other cell types, we carried out the same experiments as shown in Figure 6, but we used a T-lymphocyte cell line CEM-SS, which models the natural target of HIV-1. However, instead of using synchronized cells, we used asynchronized cells that we normally grow in the laboratory. As shown in Figure 7A, CEM-SS cells transduced with the Adv control virus showed normal cell cycle profile, i.e., remained predominantly in G1 (2N) phase of the cell cycle over a period of 96 hours; however, a very strong G2 cell population (4N) was seen in the Adv-Vpr transduced cells 48 hours after viral transduction. Noticeably, a small increase of 8N DNA was also observed 72 to 96 hours after the adenoviral transduction. Very similar to what we have observed in HeLa cells, Vpr also induced relatively strong Chk1-Ser^345 ^phosphorylation (Figure 7B, first row, lane 2 *vs*. 1). However, only smaller reductions of Chk1-Ser^345 ^phosphorylation were seen in the Cdt1 or Cdc6-depleted CEM-SS cells compared to HeLa cells. This discrepancy was probably because we were only able to partially deplete Cdt1 and Cdc6 using the siRNAs in this particular T-cell line (data not shown). Consistent with the incomplete depletion of Cdt1 or Cdc6, a small but significant reduction of Vpr-induced G2 arrest (from 80.3% to 56.9%) was observed in the Cdt1-reduced cells; and a small reduction (9%) was seen in the Cdc6-reduced CEM-SS cells (Figure 7C).

## Discussion

In this report, by using a single cell cycle assay, we demonstrated that Vpr induces cell cycle G2 arrest through a rather unusual molecular mechanism. Vpr causes cell cycle G2/M arrest, but the triggering event occurs in the S phase of the cell cycle. Specifically we showed that the expression of HIV-1 *vpr *elicits activation of Chk1 through Ser^345 ^phosphorylation, which coincides with the hyperphosphorylation of Cdk1-Tyr^15^, a hallmark of G2/M arrest. The S phase-specific activation of Chk1 by Vpr was verified by testing the Vpr effect in different phases of the cell cycle (Figure 1) and by siRNA-mediated depletion of Chk1 (Figure 2). The exclusive requirement of Chk1-Ser^345 ^phosphorylation for Vpr-induced G2 arrest was further confirmed by site-directed mutagenesis (Figure 2). Subsequent mechanistic analysis revealed that Vpr-induced Chk1-Ser^345 ^phosphorylation and G2 arrest are likely triggered during the onset of DNA replication, since the depletion of Cdt1 by specific siRNA significantly reduced Chk1-Ser^345 ^phosphorylation and G2 arrest induced by Vpr (Figures 6 and 7). To the best of our knowledge, the Vpr effect described here is unique in that it may represent a novel viral action for modulating host cell cycle regulation.

Even though Vpr-induced G2 arrest has been studied quite extensively (for reviews, see [65-68]), how the expression of *vpr *triggers cell cycle G2 arrest is not fully understood. Early reports suggested that Vpr triggers the activation of the cellular DNA damage or replication checkpoint controls for the G2 induction, because some of the classic checkpoint control genes such as ATR, Chk1, Hus1, Rad17 and γH2AX-Ser^139 ^phosphorylation are elevated upon Vpr production [44,45]. Indeed, one of the key resemblances of the Vpr effect to the HU/UV effect is the ATR-mediated Chk1 activation through Chk1-Ser^345 ^phosphorylation. Careful examination of this effect among these inducing agents revealed, however, some subtle differences. For instance, a specific isoform of PP2A is required for Vpr-induced Chk1-Ser^345 ^phosphorylation and G2 arrest, whereas the same PP2A is not needed for HU/UV-mediated Chk1-Ser^345 ^phosphorylation [48]. Moreover, under the same experimental conditions, neither HU nor UV-treated cells were able to pass through S phase in contrast to Vpr-expressing cells (Figure 3B). Consequently, HU/UV-treated cells stopped at the G1/S boundary while *vpr*-expressing cells went through S phase and arrested at the G2/M boundary. Thus even though Vpr and HU/UV all induce Chk1-Ser^345 ^phosphorylation, the cell cycle outcomes are quite different. These observations are strengthened by the additional findings that Vpr preferentially targets Cdc25C and possibly Cdc25B for Chk1-mediated inhibitory degradation by proteolysis (Figure 5). In contrast, HU/UV primarily promotes proteasome-mediated degradation of Cdc25A, instead of Cdc25C/B (Figure 4). Thus although Vpr and HU/UV all cause Chk1-Ser^345 ^phosphorylation, Vpr-induced Chk1-Ser^345 ^phosphorylation might be unique in that it is mediated through a PP2A-dependent process [48], which could further lead cells into the G2 phase of the cell cycle where Cdc25B and Cdc25C are primarily active and thereby being more affected relative to Cdc25A.

Unlike what we described here, prior studies including ours have shown that both UV and Vpr induce γH2AX-Ser^139 ^phosphorylation, a classic sign of DNA damage and the activation of DNA damage checkpoints [45,48]. γH2AX-Ser^139 ^phosphorylation was typically observed 48 hours after *vpr *gene expression. These observations suggest that the observed γH2AX-Ser^139 ^phosphorylation is more likely a late event induced by the *vpr *gene expression rather than the cause of Vpr-induced G2 arrest. This notion is supported by our new observation showing that no γH2AX-Ser^139 ^phosphorylation was found beyond the background level in cells transduced by Adv-Vpr in the first round of cell cycle; whereas low dose UV induces strong γH2AX-Ser^139 ^phosphorylation during the same time period (Figure 3A, row 2). Even though this observation does not exclude the possibility that Vpr may still induce a low level of DNA damage leading to G2 arrest, it nevertheless supports one of our earlier studies showing that a special isoform of PP2A, which is required for Chk1-S^345 ^phosphorylation and Vpr-induced G2 arrest, is not required for γH2AX-Ser^139 ^phosphorylation. Furthermore, depletion of γH2AX has no effect on Vpr-induced Chk1-S^345 ^phosphorylation and Vpr-induced G2 arrest [48].

The potential differences between the Vpr effect and activation of DNA checkpoint controls were also implicated by early studies from the fission yeast model showing that Vpr is still able to induce G2 arrest when those checkpoint control genes such as Rad3 (ATR/ATM) or Chk1 were depleted [46,69,70]. Since DNA checkpoint control genes are highly conserved among eukaryotes, those observations in fission yeast reinforce the idea that Vpr may induce G2 arrest through a molecular mechanism that is somehow different from activation of the classic checkpoint controls (for reviews, see [68,71]). Therefore, combining some of the early observations with the new findings described here, we conclude that Vpr induces cell cycle G2 arrest through a unique mechanism that is most likely different from the activation of DNA damage or replication checkpoint controls.

One of the most unique findings described here for Vpr-induced G2 arrest is that although Vpr arrests cells in G2 phase of the cell cycle, but the initiation event actually occurs in the S phase of the cell cycle. Mechanistically, we now show that this S phase-dependent initiation of cell cycle G2 arrest is likely triggered by cellular signaling of DNA re-replication through the DNA licensing factor Cdt1 and possibly Cdc6 (Figures 6 and 7). The replication licensing factors Cdt1 and Cdc6 are essential cellular proteins for ensuring DNA replicates only once per cell cycle in all eukaryotes [56,57]. In particular, Cdc6 functions in conjunction with Cdt1 to promote the loading of minichromosome maintenance (MCM) complex for initiation of DNA replication [58]. In fact, Cdt1 and Cdc6 are mutually dependent upon each other for MCM loading and initiation of DNA replication through direct protein-protein interaction [58]. No DNA replication licensing will be initiated if Cdc6 failed to bind Cdt1 [72]. Conversely, abnormal elevation of Cdt1 and Cdc6 will lead to DNA re-replication [57,63], which causes Chk1-Ser^345 ^phosphorylation [60]. Interestingly, however, overexpression of either Cdt1 or Cdc6 alone does not induce detectable re-replication [63], further confirming the synergistic relationship between these two proteins. Thus, Cdt1 and Cdc6 are normally tightly regulated during the cell cycle and are rapidly inhibited or degraded upon onset of DNA replication by various mechanisms to prevent re-replication (for a recent review, see [59]).

Our data described here suggest that Cdt1 might be one of the primary contributing factors with the possible contribution of Cdc6 to Vpr-induced Chk1-Ser^345 ^phosphorylation and G2 arrest. This notion is certainly supported by our observations that depletion of Cdt1 and/or Cdc6 by siRNA significantly reduced Vpr-induced Chk1-Ser^345 ^phosphorylation (Figure 6B, Figure 7B) and cell cycle G2 arrest (Figure 6C, Figure 7C). Moreover, depletion of both Cdt1 and Cdc6 at the same time showed no additional reduction of Chk1-Ser^345 ^phosphorylation (data not shown) indicating that Cdt1 and Cdc6 are indeed working in the same pathway for the Chk1 activation. It is unclear for the moment why depletion of Cdc6 gave rise to less reduction of Vpr-induced Chk1-Ser^345 ^phosphorylation (Figure 6B-a, lane 4 *vs. *6; Figure 7B) and G2 arrest (Figure 6C, Figure 7C). One possibility is that the Cdt1 activity is regulated, besides Cdc6, by additional mechanisms including the inhibitory binding of geminin and Cul4-DDB1-Cdt2-mediated protein degradation (for a review, see [59]). It is thus likely that Cdc6 participates only partially in regulating Cdt1 for Vpr-induced DNA re-replication. Consistent with the involvement of Cdt1 and Cdc6 in the Vpr effect, Vpr induces the accumulation of increasing DNA ploidy over time [[54,55]; (Figure 6A-b, Figure 7A)] implicating DNA re-replication. Noticeably, however, only a relatively low level of 8N DNA accumulation was observed. Since a small increase of Cdt1 activity could induce DNA re-replication [57,63] leading to Chk1-Ser^345 ^phosphorylation [60], and since the depletion of Cdt1 diminishes Vpr-induced Chk1-Ser^345 ^phosphorylation (Figure 6B, Figure 7B), it is conceivable that the low level of DNA re-replication triggered by Vpr is probably sufficient to induce Chk1-Ser^345 ^phosphorylation and cell cycle G2 arrest. Verification of this possibility is certainly warranted in future studies. It should be mentioned that the effects of Vpr on Chk1-Ser^345 ^phosphorylation and Cdt1 or Cdc6 were shown here in two different cell types (HeLa and CEM-SS), which suggest a general effect of Vpr on these cells. Noticeably, however, a much reduced impact of Vpr was seen in the CEM-SS cells than in HeLa cells. This discrepancy is probably due to the fact that Cdt1 or Cdc6 was only partially reduced in CEM-SS cells; whereas nearly complete depletion of these two proteins was obtained in HeLa cells. In addition, these effects were tested in synchronized HeLa cells, which could show cell cycle-specific effect; while when asynchronized CEM-SS cells were used Vpr may have little or no effect in other cell cycle phases except the S-G2 phases. Our future investigation will attempt to resolve these differences. Intriguingly, a recent paper [73] reported that a HBV viral protein X (pX) also induces partial polyploidy and DNA re-replication; but it promotes Cdt1 activity through increase the Cdt1-to-germini ratio. Even though HBV pX and HIV Vpr do not otherwise share the same molecular mechanism of actions, the fact that two distinct viral proteins are both affecting the same cellular target as Cdt1 may imply some potential underlying similarities between these two viruses during host-pathogen interactions.

In summary, we have shown in this study that Vpr interferes with host cell cycle regulation through a very distinctive molecular mechanism that could be characteristically different from the cell cycle DNA checkpoint controls. Even though the biologic and virologic significance of this unique viral action in HIV-1 infected cells is not fully understood, in-depth study of the molecular mechanism underlying Cdt1/Cdc6-mediated induction of cell cycle G2 arrest by Vpr through an S-mediated cellular event(s) could have broad impact toward our understanding of the basic host cell cycle regulation and HIV biology.

## Methods

### Cell Line, Cell Cycle Synchronization and Adenoviral Transduction

HeLa cells were grown in Dulbecco's modified Eagle's medium (DMEM) (Cellgro) and CEM-SS cells were grown in RPMI 1640 medium supplemented with 10% heat-inactivated fetal calf serum (FCS, Invitrogen) and 100 unit/ml of penicillin/streptomycin. HeLa cells were synchronized to the G1/S boundary of the cell cycle using a previously described double thymidine (DT) block method [49]. Synchronized mitotic cells were obtained following treatment with Nocodazole for 20 hrs (100ng/ml, Sigma) [50]. To induce DNA checkpoints, cells were treated either with HU (10mM) or UV (10 sec at the dose rate of 3 J/m^2^) immediately after cell release from the DT block. Similarly, synchronized cells were also transduced immediately after release of the DT block with the adenoviral vector control (Adv) or with Vpr (Adv-Vpr) by using MOI of 1.0 as we described previously [74,75]. The Adv and Adv-Vpr vectors were provided by Dr. Ling-Jun Zhao (St. Louis University, St. Louis, MO) and have been described previously [74-76]. Cells were harvested at the indicated time for further analysis.

### Plasmids and Site-directed Mutagenesis

A mammalian expression plasmid pEGFP-Chk1 that expresses wild type (WT) Chk1 [77] was used as a template to construct the siRNA-resistant WT Chk1 (siR-Chk1) and its mutant derivatives. Specifically, two rounds of PCR were used to construct the siR-Chk1, i.e., the modified *Chk1 *gene transcripts produce the same WT Chk1 proteins, but they cannot be depleted by the siRNA normally used to deplete endogenous Chk1 (Cat. No. 1024702, Qiagen). Briefly, the pEGFP-Chk1 WT was used as a template in the first round PCR with forward primers 5'-CAT GGT CCT GCT GGA GTT CGT G-3' (P1) and reverse primers 5'-CTT AAT ATT TTC GGG GCA ATC CAC TGC TCT TTT CAT ATC TAC AAT CTT CAC-3' (P2) to generate left side of the WT *Chk1 *sequence, and with forward primers 5'-GTG AAG ATT CTA CAT ATG AAA AGA GCA GTG GAT TGC CCC GAA AAT ATT AAG-3' (P3) and reverse primers 5'-ACT GCA GAA TTC GAA GCT TGA GCT CGA ACG GG-3' (P4) to generate the right side of the WT *Chk1 *sequence with 51 base pairs overlapping. After isolating each of the PCR products using agarose gel elution kit, the second round of PCR was conducted with each of first round PCR product and the P1 and P4 primers. The PCR product was isolated and digested with *Xho*I/*EcoR*I restriction enzymes. The digested products were then sub-cloned at the same restriction sites into the parental plasmid pEGFP-C1. Site-directed mutagenesis of the siRNA-resistant Chk1-S^345 ^phosphorylation-site was carried out by the using same procedure as described above with specific nucleotide mutant built in the primers. In this case, the serine residue at position 345 was changed to alanine by using the siR-Chk1-carrying plasmid as template, which results in siRNA-resistant mutant Chk1-S345A (siR-Chk1-S345A). All mutant constructs were confirmed by nucleotide sequencing.

### Cell Cycle Analysis

At the indicated time, cells were collected by trypsinization. Cells were then washed twice with 2 ml of 5 mM EDTA/PBS and centrifuged at 1,500 rpm. After resuspension in 1 ml 5 mM EDTA/PBS, cells were fixed with 2.5 ml of 95-100% cold ethanol and kept at 4°C overnight. After centrifugation, fixed cells were washed twice with 2 ml of 5 mM EDTA/PBS and centrifuged at 1,500 rpm. After resuspension in 0.5 ml PBS, cells were incubated with RNase A (50 μg/ml) at 37°C for 30 minutes and then at 0°C with addition of propidium iodine (PI, 10 μg/ml) for 1 hour. Cells were then filtered prior to analysis of DNA content by FACScan flow cytometry (Becton Dickinson). The cell cycle profiles were modeled by use of the ModFit software (Verity Software House, Inc.).

### SiRNA Transfection

Specific siRNA duplex against endogenous Chk1 "Chk1 siRNA" (Cat. No. 1024720), the FlexiTube siRNA against Cdt1 (Cat. No. SI04159477 and SI04142250) and the control non-silencing siRNA (Cat. No. 1022083) were purchased from Qiagen (Valencia, CA). The siGenome Smartpool siRNA against Cdc6 (Cat. No. M-003233-02) was purchased from Dharmacon (Chicago, IL). The siRNA mixtures were transfected at a concentration of 10 nM into approximately 5 × 10^5 ^dividing HeLa or CEM-SS cells by using 8 μl of Lipofectamine RNAiMAX following manufacturer's instructions (Invitrogen). Measurement of transfection efficiency of siRNAs by using Rhodamine labeled siRNA indicated >90% transfection efficiency.

One of the technical challenges for Cdt1 depletion by siRNA is that we cannot test prolonged depletion effect of Cdt1 because it causes cell death [[78,79]; our unpublished data]. However, an early study showed that depletion of Cdt1 after first few rounds of DNA replication does not affect cell viability and ongoing cell cycle profile [78]. Thus Cdt1 or Cdc6 was depleted and tested in our experiments as follows. Briefly, HeLa cells were treated with 2 mM thymidine for 18 hrs (first thymidine block) then thymidine was removed by washing with PBS three times. Specific siRNA against Cdt1, Cdc6 or control siRNA was then added with fresh media for 8 hrs. The HeLa cells were further synchronized to G1/S boundary with the second thymidine block for 16 hours. The Cdt1 or Cdc6 depletion effect was measured at 5 or 11 hours after the DT release and transduction with Adv-Vpr. For CEM-SS cells, asynchronized CEM-SS cells were pretreated with Cdt1, Cdc6 or Ctr siRNA, and then were transduced with Adv or Adv-Vpr 24 hours after addition of siRNAs. Cells were then harvested 48 hours post-transduction for further analyses.

### Antibodies

Rabbit monoclonal anti-phospho-Chk1-Ser^345 ^(133D3) antibody was purchased from Cell Signaling Technology, Inc (Danvers, MA). Mouse monoclonal anti-Chk1 (G-4), mouse monoclonal anti-Cdc25A (F-6) and rabbit polyclonal anti-Cdt1 antibodies were purchased from Santa Cruz Biotechnology, Inc (Santa Cruz, CA). Mouse monoclonal anti-Cdc25B (DCS.162.2) antibody was from EMD Chemicals, Inc (Gibbstown, NJ). Mouse monoclonal anti-Cdc25C (TC-15) antibody and mouse monoclonal anti-phospho-Histone γ-H2AX (Ser139) were from Upstate, Inc (Lake Placid, NY). Mouse monoclonal anti- Cdc6 (DCS-180) and mouse monoclonal anti-β-actin (AC-15) antibodies were from Sigma-Aldrich, Inc. Goat anti-mouse IgG (H+L) HRP conjugate and goat anti-rabbit IgG (H+L) HRP conjugate secondary antibodies from BioRad, Laboratories (Hercules, CA), and rabbit polyclonal anti-Vpr serum was custom generated through the Proteintech Group, Inc (Chicago, IL).

### Western Blotting

Cells were lysed with lysis buffer (50 mM Tris, pH7.5, 150 mM NaCl, 2 mM EDTA, 1% Triton X-100) on ice for 30 minutes, and the debris was removed by centrifugation at 13,000 rpm for 1 minute. The protein concentrations of supernatants were measured by BCA protein assay kit (Pierce). After boiling, 50 μg of protein were loaded on Criterion Precast Gels (BioRad) for electrophoretic separation. Proteins were transferred to the Trans-blot^® ^Nitrocellulose membranes and blocked with 5% skim milk in TBST buffer (10 mM Tris, pH 8.0, 150 mM NaCl, 0.1% Tween 20) for 1 hour at room temperature. Primary antibodies were then applied overnight at 4°C. After washing 3 times in TBST for 10 minutes each time, the membranes were incubated with secondary antibody for 1 hour at room temperature. Membranes were washed again, and proteins were detected with Supersignal^® ^West Dura Chemiluminescent Substrate (Pierce, Rockford, IL).

To quantify the intensity of protein of interest, densitometry was used to quantify the protein band and compared to either the protein loading control such as β-actin or the same protein at the baseline.

## Competing interests

The authors declare that they have no competing interests.

## Authors' contributions

GL carried out most of the experiments and participated in the experimental designs, data analyses and preparation of the manuscript; HUP constructed the siRNA-resistant pEGFP-Chk1 and Ser345A mutant plasmids; DL assisted in preparation and adenoviral experiments; RYZ oversaw the entire project including experimental designs, data analyses and preparation of the manuscript. All authors read and approved the final manuscript.
